# Development of Lidocaine-Loaded Dissolving Microneedle for Rapid and Efficient Local Anesthesia

**DOI:** 10.3390/pharmaceutics12111067

**Published:** 2020-11-09

**Authors:** Huisuk Yang, Geonwoo Kang, Mingyu Jang, Daniel Junmin Um, Jiwoo Shin, Hyeonjun Kim, Jintae Hong, Hyunji Jung, Hyemyoung Ahn, Seongdae Gong, Chisong Lee, Ui-Won Jung, Hyungil Jung

**Affiliations:** 1 JUVIC Inc., No. 208, Digital-ro 272, Guro-gu, Seoul 08389, Korea; hsyang@juvicbio.com (H.Y.); gwkang@juvicbio.com (G.K.); mgjang@juvicbio.com (M.J.); hjkim@juvicbio.com (H.K.); jthong@juvicbio.com (J.H.); hjjung@juvicbio.com (H.J.); hmahn@juvicbio.com (H.A.); sdgong@juvicbio.com (S.G.); 2 Department of Biotechnology, Building 123, Yonsei University, 50 Yonsei-ro, Seodaemun-gu, Seoul 03722, Korea; danny0619@yonsei.ac.kr (D.J.U.); jiwooshin@yonsei.ac.kr (J.S.); lchs0625@yonsei.ac.kr (C.L.); 3 Department of Periodontology, Research Institute for Periodontal Regeneration, Yonsei University College of Dentistry, 50 Yonsei-ro, Seodaemun-gu, Seoul 03722, Korea

**Keywords:** dissolving microneedle, local anesthesia, lidocaine, transdermal drug delivery

## Abstract

Lidocaine is a local anesthetic agent used in the form of injection and topical cream. However, these formulation types have limitations of being either painful or slow-acting, thereby hindering effective and complete clinical performance of lidocaine. Dissolving microneedles (DMNs) are used to overcome these limitations owing to their fast onset time and minimally invasive administration methods. Using hyaluronic acid and lidocaine to produce the drug solution, a lidocaine HCl encapsulated DMN (Li-DMN) was fabricated by centrifugal lithography. The drug delivery rate and local anesthetic quality of Li-DMNs were evaluated using the pig cadaver insertion test and Von Frey behavior test. Results showed that Li-DMNs could deliver sufficient lidocaine for anesthesia that is required to be utilized for clinical level. Results from the von Frey test showed that the anesthetic effect of Li-DMNs was observed within 10 min after administration, thus confirming fast onset time. A toxicity test for appropriate clinical application standard was conducted with a microbial limit test and an animal skin irritation test, showing absence of skin irritation and irritation-related microorganisms. Overall, Li-DMN is a possible alternative drug delivery method for local anesthesia, meeting the requirements for clinical conditions and overcoming the drawbacks of other conventional lidocaine administration methods.

## 1. Introduction

Several clinical procedures, including venipuncture and dermatological procedures, are associated with pain, which causes anxiety and stress to the patient [1]. Accordingly, various anesthetic methods, such as general, regional, and local anesthesia, have been developed to relieve procedural pain by suppressing the transmission of signals to the nervous system [2,3]. These methods differ based on the area affected by the anesthetic agents, varying from the whole body or central nervous system to small area of the skin. Local anesthesia has the advantage of being safer compared to other anesthetic methods due to low body stress, low drug dosage, and fast recovery, and is widely used in minor dental and dermatological surgeries and biopsies. Lidocaine, also known as xylocaine, is a widely used local anesthetic agent for the management of acute and chronic pain [4]. To exert the local anesthetic effects, anesthetic agents need to be transported to the nervous system through the skin barrier. Hypodermic needle injection is considered as the main method for drug administration [5]. However, using syringes has several disadvantages, including pain, risk of infection, and induction of trypanophobia [6,7]. In addition, the use of hypodermic needles requires trained medical experts [5,6]. To avoid these limitations of hypodermic injection, topical anesthetics have been developed in the form of creams, gels, and sprays [8], and have been demonstrated as effective superficial anesthetic agents [9]. Nevertheless, conventional topical anesthetic creams have slow onset time (up to 60 min) owing to difficulty in crossing the skin barrier, which limits the maximum molecular weight of available anesthetic agents to 500 Dalton [10,11,12]. To overcome this limitation, skin anesthesia using a combination of ultrasound pretreatment and iontophoresis can reduce the onset time to less than 10 min [12]. However, skin irritation has been reported during electrical treatment due to physiological changes in the skin [13]. Therefore, there is a need for a method capable of efficiently delivering anesthetics across the physical barrier of the skin for a fast onset time.

Microneedle (MN) is a novel transdermal drug delivery system that can deliver drugs across the stratum corneum, the outermost physical barrier of the skin, in a minimally invasive manner [14]. Considering the potential of MN to overcome the skin barrier and reduce onset time by delivering the drug directly into the skin, application of MN for the delivery of lidocaine has been introduced. First, a coated microneedle (CMN) was introduced by coating a polymer-based microneedle with lidocaine to deliver lidocaine within 4 min of application [1]. However, the amount of coated drug was insufficient due to the limited surface area of MN, and drug delivery with CMN had issues such as low biocompatibility and generation of biohazardous waste [10]. Hence, a novel type of drug delivery method that can overcome the aforementioned drawbacks is needed. Dissolving microneedles (DMN) is regarded as an alternative to CMN and can be used to address these problems [10]. DMN, which is fabricated using a mixture of biodegradable polymers and bioactive materials [14], can deliver its contents by dissolving in body fluids after skin penetration, and has a rapid onset time of 10 min [10,15]. Therefore, lidocaine-encapsulated DMNs have been developed for enhanced delivery of local anesthetics [15]. While delivery of lidocaine using DMN has a rapid onset time compared with conventional local anesthetics, studies on the clinical application of lidocaine encapsulated DMNs, such as mechanical strength evaluation, assessment of insertion success rate, quantitative analysis of lidocaine content, and skin irritation test have not been conducted.

In this study, we propose a lidocaine HCl-encapsulated DMN (Li-DMN) that can reach the nerve cells of the dermis. This Li-DMN could provide a rapid onset time of 10 min compared with the widely used conventional topical anesthetics, including EMLA cream. In addition, to meet the clinical application standard, we analyzed whether the Li-DMN patch contained a sufficient amount of lidocaine to exert a local anesthetic effect. Moreover, we introduced a novel evaluation method to visualize the insertion success rate of DMNs and verify whether Li-DMNs are suitable for practical use. The safety issue test, required by the Korea Food and Drug Administration (KFDA) for drugs, was conducted to verify the practical application potential for Li-DMNs. Through these studies, we demonstrated that the Li-DMN patch was biocompatible, could deliver local anesthetic more rapidly compared to conventional topical local anesthetics, and was suitable for practical use; and thus providing practical guidelines for the use of Li-DMNs for further human-targeted applications. This novel type of local anesthetic drug delivery system has the potential to deliver local anesthesia conveniently and efficiently, which will further facilitate access to the anesthetic market.

## 2. Materials and Methods

### 2.1. Fabrication of Lidocaine HCl-Encapsulated DMN (Li-DMN)

Hyaluronic acid (HA) (Bloomage Freda Biopharm Co. Ltd., Jinan, China) was used as the backbone polymer of the Li-DMNs. To prepare a solution of the drug and polymer, lidocaine hydrochloride (Mahendra Chemicals, Gujarat, India) was blended with HA in distilled water and homogenized using a paste mixer (PDM-300C, KM TECH Co. Ltd., Icheon, Korea). Then, the Li-HA solution was dispensed on a general-purpose polystyrene film using a robotic dispenser (ML-5000X, Musashi Engineering, Inc., Tokyo, Japan) in a hexagonal array. The array contained 61 droplets that could potentially have 2.11 mg of lidocaine content in a single array. The mixture droplets were centrifuged for 10 s at 3095× *g* rpm to form a DMN shape, using a centrifugal lithography method, which is a technique for fabricating DMNs by centrifugal force [16].

### 2.2. Evalaution of the Physical Properties of Li-DMN

To assess the morphological properties of Li-DMNs, microscopic images were acquired under a bright field microscope (M165FC, Leica Camera AG, Wetzlar, Germany) and a scanning electron microscope (JSM-7610F Plus, JEOL Ltd., Tokyo, Japan). The mechanical strength of Li-DMNs was analyzed using a displacement force machine (Z0.5TN, Zwick Roell Inc., Ulm, Germany). Each DMN was separated from the Li-DMN patch and set on the stage of the machine, and then the probe moved vertically downward at a speed of 3.6 mm/min. Subsequently, the axial force was recorded when the probe pressed the DMN and the fracture of DMN occurred.

### 2.3. Elution Assessment of Li-DMN

Elution tests of Li-DMNs were conducted according to the guidelines of the Korean Pharmacopia, using a dissolution tester (DIS 600i, Copley, Nottingham, United Kingdom). Phosphate-buffered saline (PBS) was put into the dissolution tester, maintained at a temperature of 37 °C and a rotation speed of 100 rpm. Li-DMN patches (*n* = 6) were dissolved in 900 mL of PBS to obtain the test sample. Ten mL of the test sample was collected after 10 min and evaluated.

### 2.4. Evalaution of Skin Insertion Ability of Li-DMN on Pig Cadaver Skin

The skin insertion ability of Li-DMNs was evaluated by applying Li-DMN patches, composed of 61 DMNs, on pig cadaver skin (Cronex, Hwaseong, Korea) for 10 min. Li-DMN patches were applied by applicator with compression force of 20 to 25 N. Back site of pig cadaver skin with 1 mm thickness was thawed in a water bath at 37 °C for more than 30 min and dried at room temperature for 10 min. To obtain the insertion rate of Li-DMNs, each MN was observed under a brightfield microscope after application on pig cadaver skin, and the insertion rate was analyzed by calculating the number of dissolved DMNs post application. A MN with the tip dissolved and the basement left without dissolving was considered a successful insertion. A MN with a curved tip was considered as failure insertion. An insertion success was marked with a green field, while a failure was marked with a red field. Each of the 61 DMNs in a patch was observed to determine whether the insertion quality was successful or not.

### 2.5. In-Vivo Transdermal Delivery of Li-DMN

All animal experiments were approved by the Institutional Animal Care and Use Committee (IACUC) at Korea Confomity Laboratories (KCL), and the experimental procedures were executed in accordance with the guidelines of the IACUC at KCL. Male Sprague-Dawley rats (eight weeks old) were kept at animal facilities for one week before the experiments and were maintained at a temperature of 22 ± 3 °C, with a 12 h light–dark cycle. The animals were provided free access to food and water. Body weight was measured the day before the experiment, and only animals weighing ± 20% of the average body weight were used in the experiment. To evaluate the transdermal delivery of Li-DMNs, quantification of Li-DMN in skin was performed. The animals were divided into two groups: Li-DMN group and EMLA 5% cream group (*n* = 5 per group). The Li-DMNs were applied to the poll of rats for 10 min after removing hair, while the EMLA 5% cream was applied for 60 min. For the application of the Li-DMN patch and EMLA 5% cream, skin tissue was obtained using an 8-mm disposable biopsy punch (Kai Inudstries Co., Ltd., Tokyo, Japan). Homogenization and elimination of skin tissue were done to obtain specimens for liquid chromatography-mass spectrometry (LC-MS). Pharmacokinetic parameters of Li-DMN and EMLA 5% were determined by measuring the Li-HCl concentration in serum over time. Blood sampling was performed hourly after administration and the concentration of lidocaine in blood was analyzed using liquid chromatography-tandem mass spectrometry (LC-MS/MS) method. LC: 1290 Infinity (Agilent, Santa Clara, CA, USA), MS/MS: 6460 Triple Quadruple (Agilent, USA) [17]. The maximum drug concentration observed (C_max_) value and time of maximum drug concentration observed (T_max_) values were acquired by LC-MS/MS analysis. The area under the curve (AUC) value was calculated using Phoenix^TM^ WinNolin 6.3 (Pharsight, Mountain View, CA, USA). Project identification code: NT19-00064 (31 March 2020). 

### 2.6. Behavioral Assessment of the Rats after Application of Li-DMN

Male Sprague-Dawley rats (six weeks old) were adapted to animal facilities one week before the experiments. After one week of adaptation, the animals were divided into two groups: Li-DMN patch application and control patch application. Both Li-DMN and control patches were applied to the paws of the rats for 10 min. The von Frey test, which measures the withdrawal threshold of mice using von Frey filaments of different intensities, was performed before and immediately after detachment of the patches in both the groups, and the experiment was conducted again after 10 min, 20 min, and 30 min. The foot of the mouse was pressed against the von Frey filament until the filament bent and held for 3 s or until the mouse moved its foot. The reaction of the mouse determined the threshold of force required for nociception, also indicating anesthetic quality. Project identification code: 20-KE-159 (22 April 2020). 

### 2.7. Toxicity Test of Li-DMN

Microbial limit tests for *Escherichia coli*, *Salmonella enterica* supsp., *Pseudomonas aeruginosa*, and *Staphylococcus aureus* were conducted using a Li-DMN patch, in accordance with the guidelines of the Korean Pharmacopia. Li-DMN patches were dissolved in a buffered sodium chloride-peptone solution (pH 7.0, 90 mL). Next, the test solution was prepared by adding 10 mL of the solution to 90 mL of tryptic soy broth, which was then cultured at 30–35 °C for 18–24 h. For the detection of *E. coli*, the test solution (1 mL) was mixed with MacConkey broth (100 mL) and cultured at 42–44 °C for 24–48 h. Next, the cultured test solution was streaked on MacConky agar and cultured at 30–35 °C for 18–72 h to verify colony formation. The test solution was mixed with Rappaport Vassiliadis salmonella enrichment broth and streaked on xylose lysine deoxycholate agar for the detection of Salmonella. For the detection of P. aeruginosa colony formation, the test solution was streaked on cetrimide agar without adding any media. In addition, for the detection of S. aureus, the test solution was streaked on mannitol salt agar without mixing with any other media.

To prepare the sample solution for the limit test of sulfate, five Li-DMN patches, each containing 2 mg of Li-HCl, were dissolved in distilled water (10 mL). Standard solutions for comparison were made by adding sulfuric acid (0.020 mol/L, 0.010 mL) to distilled water (10 mL). The limit test was conducted 10 min after adding hydrochloric acid (3 mol/L, 1 mL) and barium chloride solution (3 mol/L, 1mL) to both the test solution and standard solution.

### 2.8. Skin Irritation Test of Li-DMN

New Zealand white rabbits were adapted to animal facilities one week before the experiments. After adaptation, only those rabbits that weighed more than 2.0 kg and had no skin disorder were selected for the experiment. Dorsal hair of the rabbits was shaved (15 × 15 cm) 24 h before the experiments, and divided into 4 sections: two abraded skin sections and two intact skin sections. The abraded skin sections were induced by scraping the stratum corneum of dorsal skin with knives. Li-DMN patches were applied on intact skin and abraded skin for the experimental group, and gauze with saline solution was applied to the other intact skin and abraded skin for the control group. After 24 and 72 h post application, skin irritation evaluation was conducted according to the Draize primary irritation index. Project identification code: GT20-00006 (8 June 2020). 

## 3. Results and Discussion

### 3.1. Design of Li-DMN

**Figure 1:**
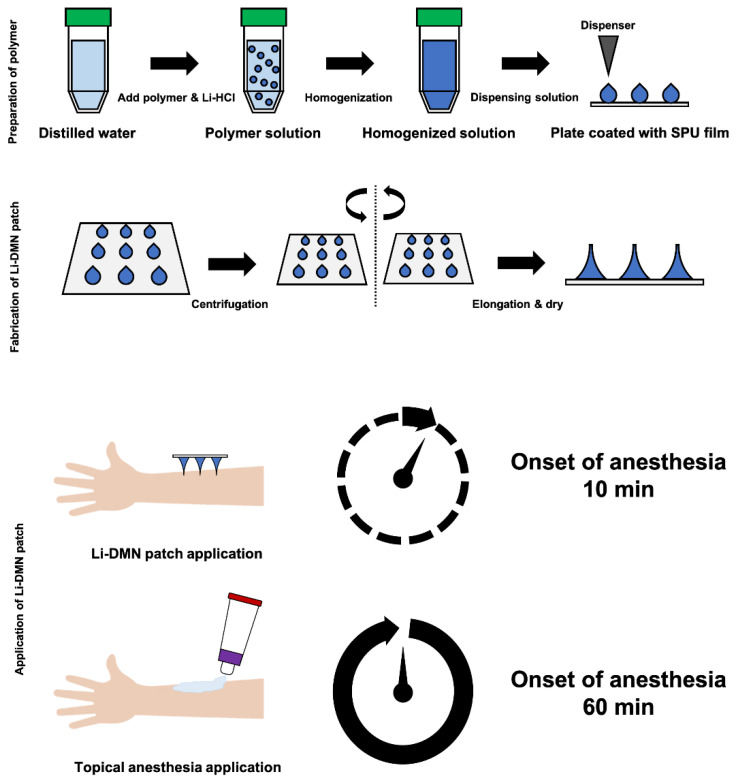
Schematic representation of the designing of lidocaine HCl-encapsulated DMN (Li-DMN). Top, preparation of polymer solution for fabrication of Li-DMN. Middle, fabrication of Li-DMN by centrifugal lithography. Bottom, application of Li-DMN. Li-DMN was designed to deliver lidocaine in 10 min for rapid local anesthesia.

During the designing stage of DMN for the delivery of Li-HCl, HA was selected as the backbone material because it is biodegradable, biocompatible, and mechanically stable [18]. In addition, Solvent Casting Polyurethane (SPU), which is a widely used biomaterial owing to its outstanding mechanical features [19], was selected as the patch backing material. After dispensing the polymer solution, each patch was placed in a centrifuge to fabricate Li-DMNs by centrifugal lithography (Figure 1) [10]. Centrifugal lithography is a method of fabricating DMNs using centrifugal force, without reducing the bioactivity of the encapsulated drugs [16]. The Li-DMNs, fabricated by centrifugal lithography, were in the form of hourglass, horizontally cut in half.

### 3.2. Physical Properties of Li-DMN

**Figure 2:**
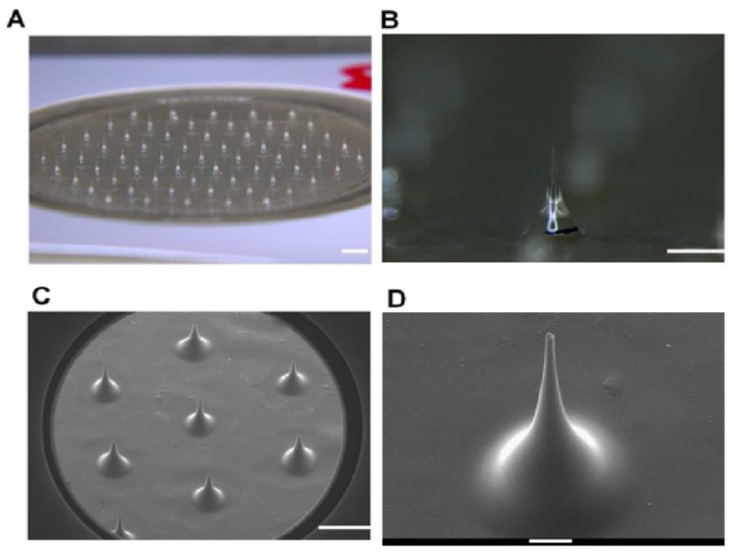
Evaluation of the physical properties of Li-DMN. (**A**) Brightfield microscopy image of Li-DMN array. Scale bar, 1 mm. (**B**) Higher magnification of brightfield microscopy image of Li-DMN array. Scale bar, 500 μm. (**C**) Scanning electron microscope image of Li-DMN array. Scale bar, 1 mm. (**D**) Higher magnification of scanning electron microscope image of Li-DMN array. Scale bar, 100 μm.

After the fabrication was completed, the morphological properties of the Li-DMN were observed using an optical microscope and a scanning electron microscope (Figure 2A–D). Microscopic observation of Li-DMN showed that the average height, base diameter and tip diameter of Li-DMN were 630.4 ± 42.2 μm, 445.5 ± 50.0 μm, and 35.3 ± 10.2 μm, respectively. Since the insertion force required to penetrate the skin is 0.058 N/needle [20], we evaluated the mechanical strength of Li-DMNs to determine whether it had sufficient mechanical strength for insertion into the skin. The mechanical force was evaluated by measuring the fracture force of each MN on the Li-DMN patch, using a displacement force machine. Through this evaluation, we determined that the mechanical strength of Li-DMNs was 0.116 ± 0.086 N (Figure S1). Moreover, this result demonstrated that the strength of Li-DMN was adequate for skin penetration, as the pushing force exerted by human thumb is at least 28.1 N, which is enough to insert the whole DMN patch [21]. Even the weakest thumb pushing force was larger than the applicator that was used in the experiments, therefore, Li-DMN patches can be used by any individuals. In addition, we performed a 10 min-assessment of lidocaine-containing Li-DMN patch to determine whether it could rapidly release a sufficient amount of lidocaine, based on the KFDA guidelines for clinical application of patch products. Results showed that Li-DMNs could release 95.6% (Figure S2) of the encapsulated lidocaine. Thus, it was confirmed that Li-DMNs could successfully insert into the skin and deliver sufficient amount of the encapsulated lidocaine for local anesthesia.

### 3.3. Transdermal Lidocaine Delivery of Li-DMN

To investigate whether the Li-DMNs were capable of transdermal drug delivery, we first conducted an in vitro pig cadaver skin penetration evaluation. The trypan blue staining method is conventionally used for the skin insertion test to confirm MN insertion [22]. However, this method does not clearly indicate how deep DMN penetrates the skin, which is important for the analysis of drug delivery via DMN because DMNs are fabricated with drug and drug delivery is dependent on the depth of DMN penetration. For these reasons, we introduced a heat map of DMN skin insertion, which visualizes the exact individual skin insertion of the DMN. Li-DMN patches were applied on pig skin for 10 min, and then removed from the skin to verify the skin penetration by observing the dissolution of Li-DMNs.

**Figure 3:**
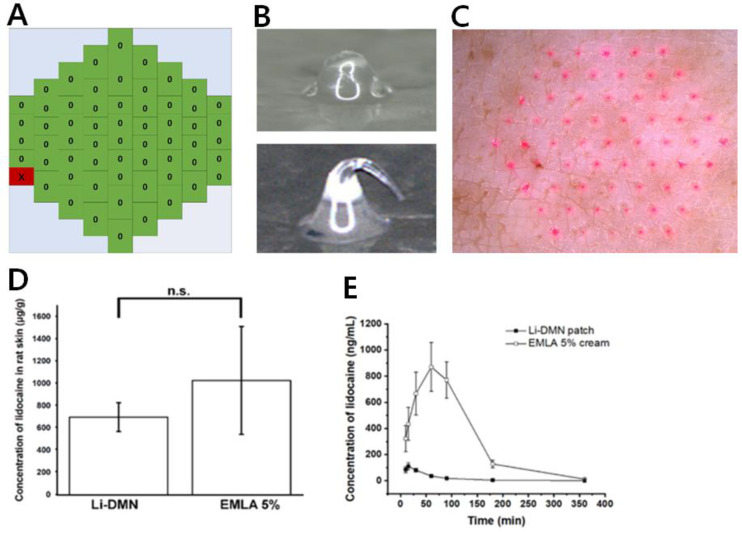
Transdermal application of Li-DMN (**A**) Heat map of Li-DMN patch after application. Green indicates successful insertion and red indicates failed insertion. (**B**) Top, representative image of successful insertion (Green in heat map). Bottom, representative image of failed insertion (Red in heat map). (**C**) Pig cadaver skin after insertion of Li-DMN with Rhodamine B as fluorescent dye. (**D**) Quantification of lidocaine concentration in Sprague-Dawley rat’s skin when applied using Li-DMN patches, and EMLA 5% cream. n.s. Not significant difference at *p* ≥ 0.05 level compared to the G1 (**E**) Pharmacokinetic profiles of Li-DMN patches and EMLA 5% cream.

In the visualized heat map, green and red fields indicated successful and failed insertion, respectively. Because Li-DMN was dissolved in the interstitial fluid of the pig cadaver skin after skin insertion, we could observe the change in the shape of Li-DMNs due to dissolution. In contrast, the Li-DMNs that failed to insert into the skin did not show this morphological change. The microscopic images of Li-DMNs, pre- and post-application, are shown in Figure 3A, as an example of green in the heat map. Compared to the original Li-DMN before insertion, the tip of Li-DMN after skin insertion was blunt and short owing to dissolution. Although the inserted DMN showed a dissolved blunt end, it did not show complete dissolution. This could be attributed to the incomplete insertion of the DMN, which is widely observed in DMN insertion [23]. This suggested that as the DMN penetrates the skin, the DMN tip enters the skin and is dissolved first, while the base remains outside the skin due to the incomplete insertion. When the Li-DMNs failed to insert into the skin, the individual DMN showed a curved shape instead of a blunt end (Figure 3B, an example of red). Because individual Li-DMNs were pressed on the skin surface without skin insertion, the tip of the DMN was forced to bend without contact with the interstitial fluid, and thus did not dissolve. For these reasons, these morphological changes in Li-DMNs demonstrate that the MN successfully inserted and dissolved into the skin after application. Additionally, Figure 3C indicates that all of the Li-DMN were inserted into the skin as the pig skin shows the traces of Li-DMN with Rhodamine B fluorescent dye. The heat map of Li-DMN showed that the average insertion rate of Li-DMNs was 99.2 ± 3.28% (Figure S3), implying that Li-DMN insertion was successfully performed (Figure S4). Penetration quality is an important feature to be considered because DMNs are drug delivery systems based on drug dissolution. Therefore, with even distribution of lidocaine in Li-DMN, better penetration quality and skin insertion rate is directly related to the amount of lidocaine delivered into the skin.

Since we loaded lidocaine not just at the tip of Li-DMN and Li-DMN did not penetrate completely into the skin, we measured the concentration of lidocaine in Sprague-Dawley rat skin to determine the delivery efficiency of Li-DMN patches, loaded with 2.11 mg of Li-HCl (Figure 3D). To confirm the rapid and efficient drug delivery for practical use, we applied Li-DMNs for 10 min and then removed them. As a reference, EMLA 5% cream with 5.75 g Li, which is a widely used transdermal local anesthetic, was applied for 60 min. The results showed that the delivery of lidocaine by Li-DMNs was not significantly different from that of EMLA 5% cream, implying that the anesthetic effect of Li-DMN was similar to that of EMLA 5% cream. In addition, in vivo pharmacokinetic evaluation was performed to verify how lidocaine changes over time after Li-DMN application (Figure 3E). These pharmacokinetic profiles indicated that Li-DMN had a lower level of lidocaine concentration in blood than the EMLA 5% cream. These data suggest that more lidocaine residue is in the skin with Li-DMN than EMLA cream, where the nervous system is located. Although further research is required to explain why lidocaine showed slow clearance in the body, one of the possible reasons could be the backbone polymer, which was mixed and delivered with lidocaine during dissolution after skin insertion. Because a high pharmacokinetic profile in the blood is unnecessary for local anesthetic effect may induce side effects like low blood pressure, we demonstrated that Li-DMN could deliver a sufficient quantity of lidocaine and had a rapid onset time with minimal concern of side effects, based on systemic circulation of lidocaine.

### 3.4. Evaluation of the Anesthetic Effect of Li-DMN

**Figure 4:**
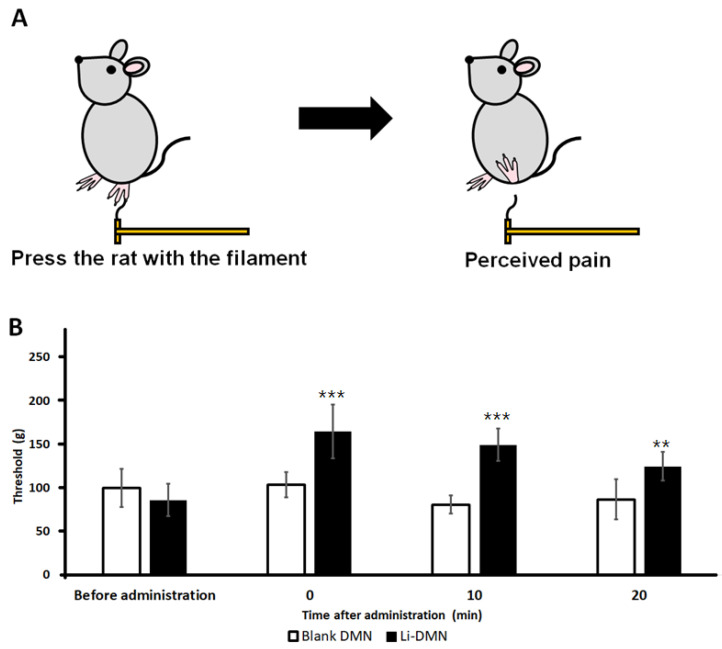
Evaluation of the anesthetic effect of control patches with no dissolving microneedles (DMNs) and Li-DMN patches. (**A**) Schematic representation of the von Frey test. (**B**) von Frey test results before administration, immediately after detachment of patches, 10 min after detachment of patches, and 20 min after detachment of patches. Both control patches and Li-DMN patches were applied for 10 min, and then detached from the rat skin. ***/** A significant difference at *p* < 0.001/*p* < 0.01 level compared with the control patches.

To assess the anesthetic effect of Li-DMNs, the von Frey test was performed using Sprague-Dawley rats (Figure 4A). Li-DMN patches were applied to the footpads of the rats for 10 min, and empty patches without anesthetics were used as control. After 10 min of application, the patches were removed from the footpads of the rats in both the groups. Immediately after removing the Li-DMN patch, the threshold (the minimum amount of force (g) required to respond to the stimulus) was 164.8 ± 31.2 g. This result was 61.0 g higher than the threshold value of controlled patch group (*p* < 0.001), indicating that an anesthetic effect appeared immediately after removing the Li-DMN patch from the footpad, thus suggesting that the rats could withstand stronger stimuli compared with those without the patch (Figure 4B). Ten minutes after the detachment of the patches, the threshold of Li-DMN group was 149.5 ± 18.4 g, which was about 68.5 g higher than the control group’s threshold of 81.0 ± 10.5 g (*p* < 0.001) (Figure 4B). Moreover, 20 min after removal of the patches, the Li-DMN group’s threshold was 124.8 ± 16.2 g, which was about 38.1 g higher than the control group’s threshold level of 86.7 ± 23.1 g (*p* < 0.01) (Figure 4B). In summary, the results of the von Frey test indicated that Li-DMN showed statistically meaningful anesthetic effects compared with those of the control group, as indicated by high lidocaine residue in the skin and pharmacokinetic profile of low shift toward blood, thereby efficiently affecting the nerve cells and avoiding possible side effects. This also implied that a local anesthetic effect was achieved without complete insertion of the Li-DMNs, as shown in Figure 3C. Despite differences in drug delivery due to incomplete insertion, anesthetic effects could still be observed since local anesthesia does not require a precise amount of drug to be delivered.

### 3.5. Toxicity Evalaution of Li-DMN

Various assessments, such as the microbial limit test, sulfate limit test, and skin irritation test, are used as criteria to determine whether the Li-DMN patch is suitable for practical application. Through the microbial limit test of the Li-DMN patch, we evaluated the presence of *E. coli*, *S. enterica subsp*., *P. aeruginosa*, and *S. aureus* in the patch, based on the KFDA guideline. The results of the microbial limit test confirmed that the aforementioned microorganisms were not present in the Li-DMN patch (Table 1). In addition, results from the limit test of sulfate showed that the Li-DMNs did not contain sulfates (Table 1), which can act as a surfactant and potentially cause skin itching and redness. Thus, the absence of sulfate content indicated that Li-DMNs may not cause skin irritation.

**Figure 5:**
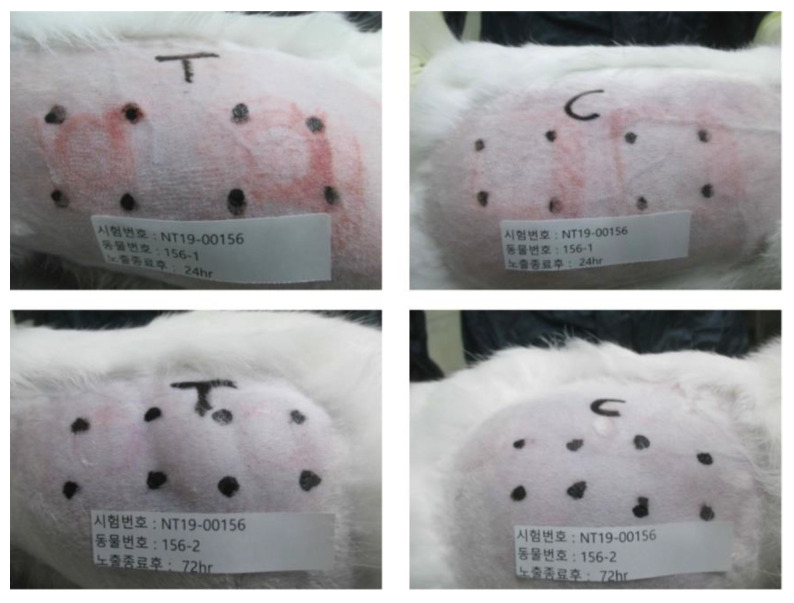
Skin irritation test of Li-DMN patch using New Zealand white rabbit. Top, skin observation after 24 h of Li-DMN application. Bottom, skin observation after 72 h of Li-DMN application. Left, abraded skin for Li-DMN application. Right, intact skin for Li-DMN application.

Additionally, we evaluated the skin reaction of New Zealand white rabbits after applying the Li-DMN patch and analyzed it using the Draize primary irritation index calculation method (Figure 5). All animals were observed for erythema, eschar, and edema formation at 24 and 72 h after the application of the Li-DMN patch, based on Draize skin irritation scoring system (Table S1) [24]. Both abraded and intact skin were used for the skin irritation test to evaluate Li-DMN under various conditions, as required by the KFDA. Both skin types were observed for erythema, eschar, and edema formation, which are the hallmarks of any potential skin irritation. The left side, which was abraded skin, showed the presence of circular erythema traces, which was due to the MN applicator during DMN administration. The intact skin on the right had less traces compared with the abraded skin. However, both the skin types did not show traces in the Li-DMN-administered part, which was at the center of four dots on the left and right sides of the skin, indicating that no wound was caused by Li-DMN itself. The result of the evaluation indicated that the primary irritation index of the Li-DMN patch was 0.5, with almost no erythema, eschar, and edema formation on both the abraded and intact skin, thus demonstrating that Li-DMN did not irritate the skin and could be suitable for clinical purposes (Table 2). These aforementioned assessments verified that Li-DMNs are not potentially harmful and are suitable for practical applications.

### 3.6. Stability Evaluation of Lidocaine

**Figure 6:**
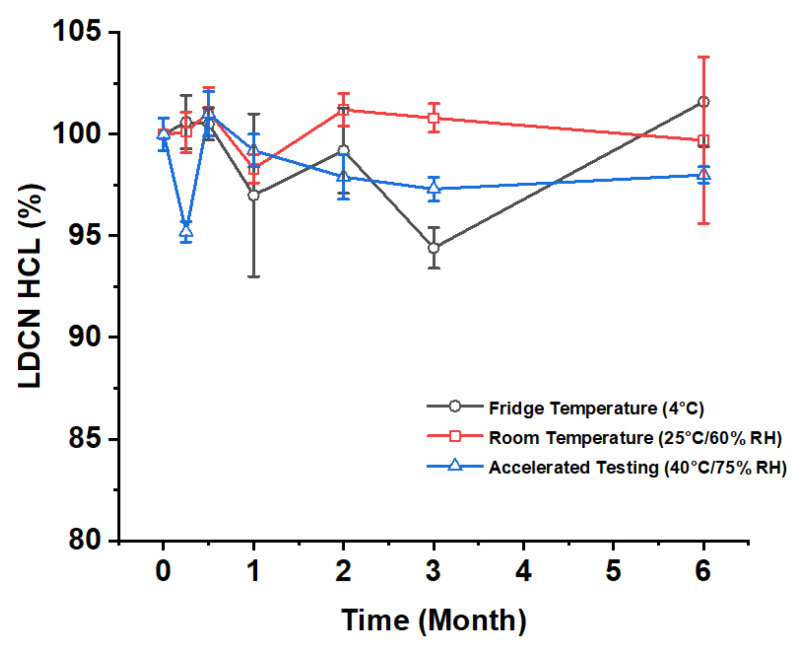
Lidocaine storage stability evaluation of lidocaine in different temperatures for 6 months. Each conditions are Fridge temperature, Room temperature, and Accelerated testing, and they are 4 °C, 25 °C/60%RH and 40 °C/75%RH, respectively. RH: Relative humidity.

Lidocaine storage stability test were conducted by using liquid chromatography-mass spectrometry. The amount of impurities in the lidocaine solution was analyzed to show the degradation of lidocaine, which in result, shows that Li-DMN can be stored for 6 months with proper storage condition (Figure 6). Even in aggressive condition, the lidocaine purity was above 90%, where the oxidation of lidocaine sample may produce side products. Still, the overall impurity percentage maintains low, indicating that lidocaine is stable even with the fabrication procedure of Li-DMN and storage of patches. Such results are particularly important for the fabricated product’s clinical application.

## 4. Conclusions

Li-DMN is an effective local anesthetic drug delivery system, with a fast onset time and an ability to deliver sufficient drug into the skin. Li-DMN can overcome the drawbacks of conventional drug delivery methods, such as injection and topical cream application, owing to fast and painless administration properties. Furthermore, it meets the requirements of clinical applications. According to the lidocaine dissolution assessment and von Frey test, Li-DMNs delivered sufficient lidocaine into the skin with a long residual time. During and after the drug delivery, the pharmacokinetic results demonstrated that lidocaine does not diffuse into the bloodstream, which can cause side effects. In addition, results from various categories of toxicity tests and skin irritation tests showed that Li-DMNs meet the requirements for clinical administration, without the possibility of containing sulfates or causing skin erythema, eschar, or edema. Overall, by meeting the requirements of the KFDA for practical clinical application, Li-DMNs have the potential to perform efficiently and can be advantageous in the field of local anesthesia.

## Figures and Tables

**Table 1:** The results of microbial limit test and sulfate limit test of Li-DMN.

**Microbial Limit Test**	**Microorganisms**	**Selective Media**	**Result**
	*Escherichia coli*	MacConkey Agar	Absent
	*Salmonella enterica subsp.*	Xylose Lysine Deoxycholate Agar	Absent
	*Pseudomonas aeruginosa*	Cetrimide Agar	Absent
	*Staphylococcus aureus*	Mannitol Salt Agar	Absent
**Limit Test of Sulfate**			**Result**
			Absent

**Table 2:** Primary irritation index (PII) results of Li-DMN.

Primary Irritation Index								
Response	Erythema and Eschar Formation				Edema			
Animal ID	Intact Skin		Abraded Skin		Intact Skin		Abraded Skin	
Time	24	72	24	72	24	72	24	72
156-1	1	0	1	0	1	0	1	0
156-2	1	0	1	0	1	0	1	0
156-3	0	0	1	0	0	0	0	0
156-4	0	0	0	0	0	0	0	0
156-5	1	0	1	0	0	0	0	0
156-6	1	0	0	0	0	0	0	0
Total	4	0	4	0	2	0	2	0
Mean	0.7	0.0	0.7	0.0	0.3	0.0	0.3	0.0
Sum	2.0							
PII	0.5							

## Supplementary Materials

The following are available online at https://www.mdpi.com/1999-4923/12/11/1067/s1, Figure S1. Fracture force test of Li-DMN. Force was applied vertically to the Li-DMN. Black arrow points to the peak indicating when the Li-DMN breakage was broken. Figure S2. Lidocaine elution test conducted using a dissolution tester. Lidocaine elution test was conducted using six different patches. Dissolution rate was obtained by comparing the difference between the solution after sterilization and after elution. Figure S3. Representative heat maps indicating skin insertion success rate using Li-DMN patches (*n* = 6). Each patch had 61 DMNs. Green box represents successful insertion of single DMN. Red box represents failed insertion of single DMN. Figure S4. Transdermal application of Li-DMN. Top, representative bright field microscopy image of lidocaine, pre (left) and post application (right). Middle, higher magnification of bright field microscopy of the same images. Bottom, representative image of Li-DMN with Rhodamine B fluorescent dye. Table S1. Primary irritation index classified by the degree of irritation.
