# Supplementary material for: Development of Lidocaine-Loaded Dissolving Microneedle for Rapid and Efficient Local Anesthesia
Source: Pharmaceutics. 2020 Nov 9;12(11):1067. doi: 10.3390/pharmaceutics12111067 (PMC7695299; doi:10.3390/pharmaceutics12111067)
Supplement: Supplementary file 1 [file pharmaceutics-12-01067-s001.pdf]

pharmaceutics

MDPI

Supplementary Materials: Development of Lidocaine-Loaded Dissolving Microneedle for Rapid and Efficient Local Anesthesia

Huisuk Yang, Geonwoo Kang, Mingyu Jang, Daniel Junmin Um, Jiwoo Shin, Hyeonjun Kim, Jintae Hong, Hyunji Jung, Hyemyoung Ahn, Seongdae Gong, Chisong Lee, Ui-Won Jung and Hyungil Jung

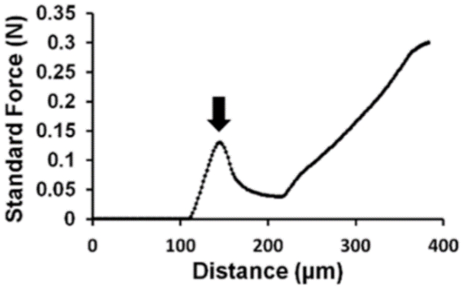

Figure S1. Fracture force test of Li-DMN. Force was applied vertically to the Li-DMN. Black arrow points to the peak indicating when the Li-DMN breakage was broken.

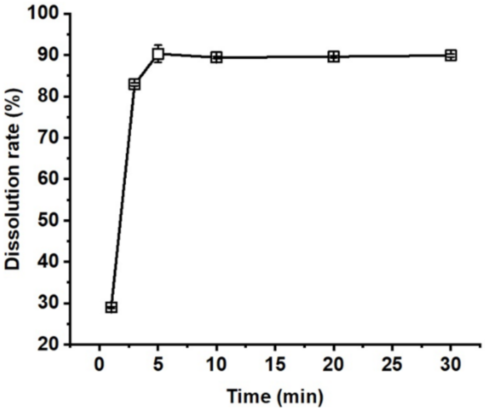

Figure S2. Lidocaine elution test conducted using a dissolution tester. Lidocaine elution test was conducted using six different patches. Dissolution rate was obtained by comparing the difference between the solution after sterilization and after elution.

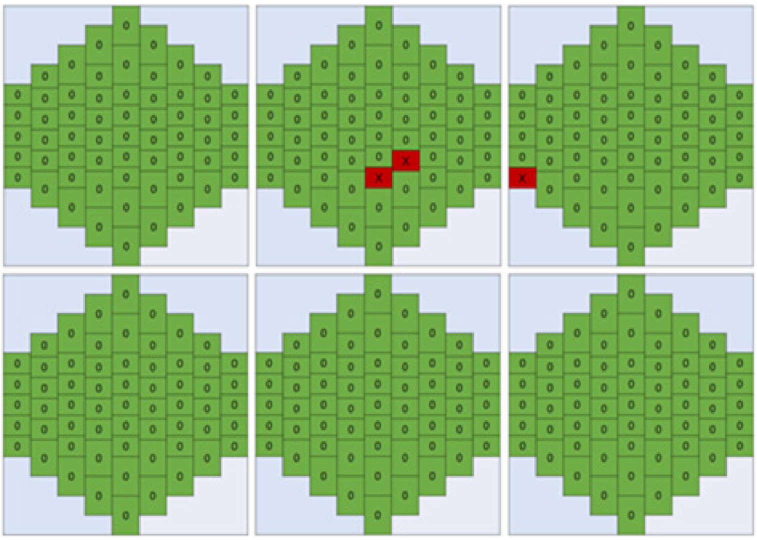

Figure S3. Representative heat maps indicating skin insertion success rate using Li-DMN patches (n=6). Each patch had 61 DMNs. Green box represents successful insertion of single DMN. Red box represents failed insertion of single DMN.

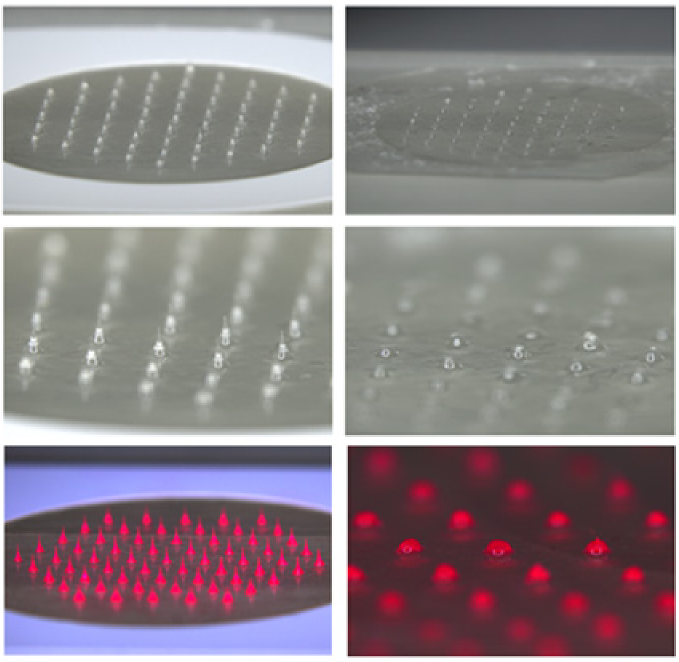

Figure S4. Transdermal application of Li-DMN. Top, representative bright field microscopy image of lidocaine, pre (left) and post application (right). Middle, higher magnification of bright field microscopy of the same images. Bottom, representative image of Li-DMN with Rhodamine B fluorescent dye.

Table S1. Primary irritation index classified by the degree of irritation.

Classification	Primary Irritation Index
No irritation	0.0 – 0.5
Slight irritation	0.6 – 2.0
Moderate irritation	2.1 – 5.0
Severe irritation	5.1 – 8.0
